# Supplementary material for: Prognostic Impact of LAG-3 mRNA Expression in Early Breast Cancer
Source: Biomedicines. 2022 Oct 21;10(10):2656. doi: 10.3390/biomedicines10102656 (PMC9599264; doi:10.3390/biomedicines10102656)
Supplement: Supplementary file 1 [file biomedicines-10-02656-s001.zip › Figure S2a.pdf]

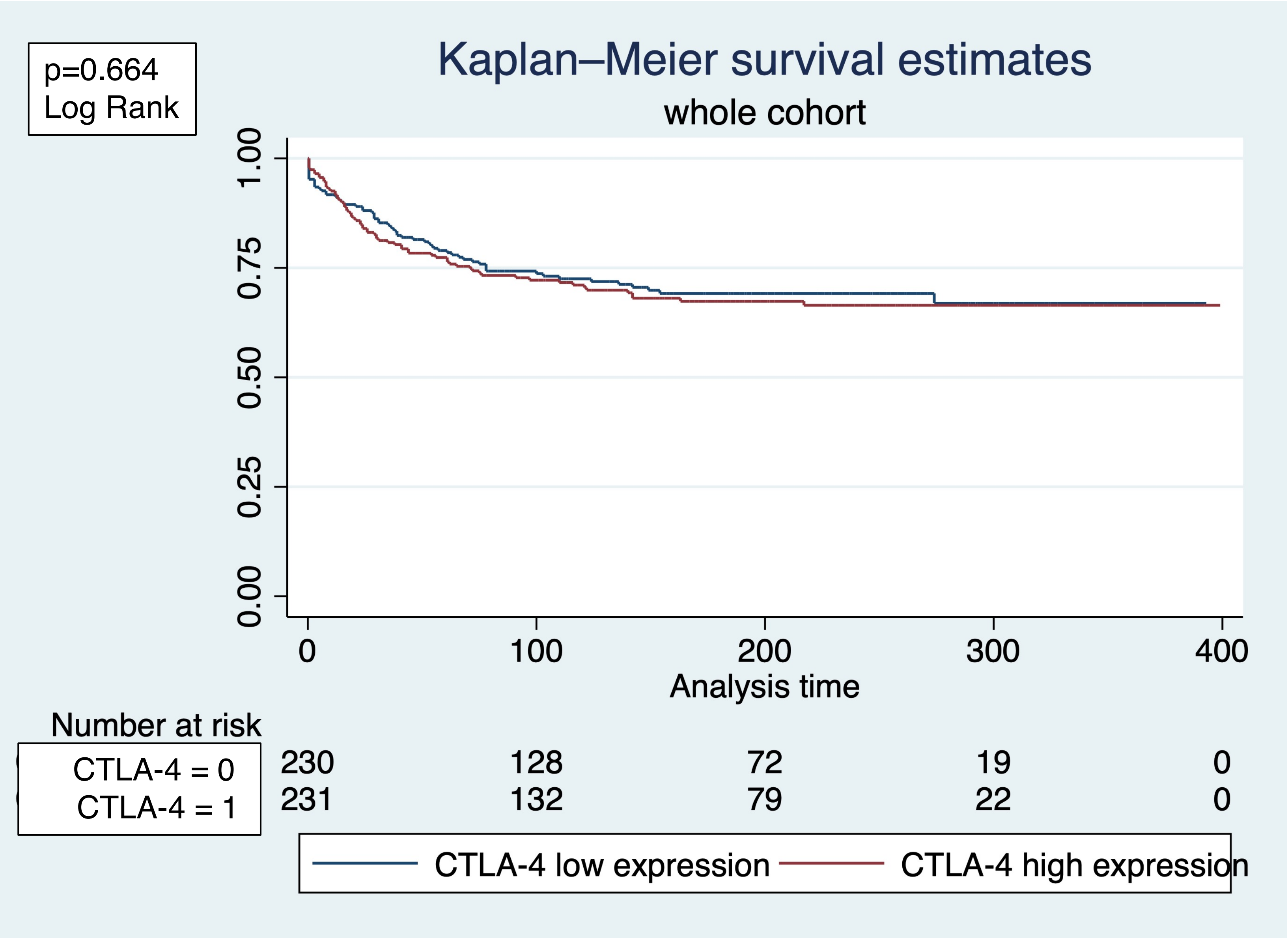
p=0.664
Log Rank
Kaplan–Meier survival estimates
whole cohort
1.00
0.75
0.50
0.25
0.00
0
100
200
300
400
Analysis time
Number at risk
CTLA-4 = 0
CTLA-4 = 1
230 128 72 19 0
231 132 79 22 0
CTLA-4 low expression
CTLA-4 high expression
