# Supplementary material for: Prognostic Impact of LAG-3 mRNA Expression in Early Breast Cancer
Source: Biomedicines. 2022 Oct 21;10(10):2656. doi: 10.3390/biomedicines10102656 (PMC9599264; doi:10.3390/biomedicines10102656)
Supplement: Supplementary file 1 [file biomedicines-10-02656-s001.zip › Figure S4b.pdf]

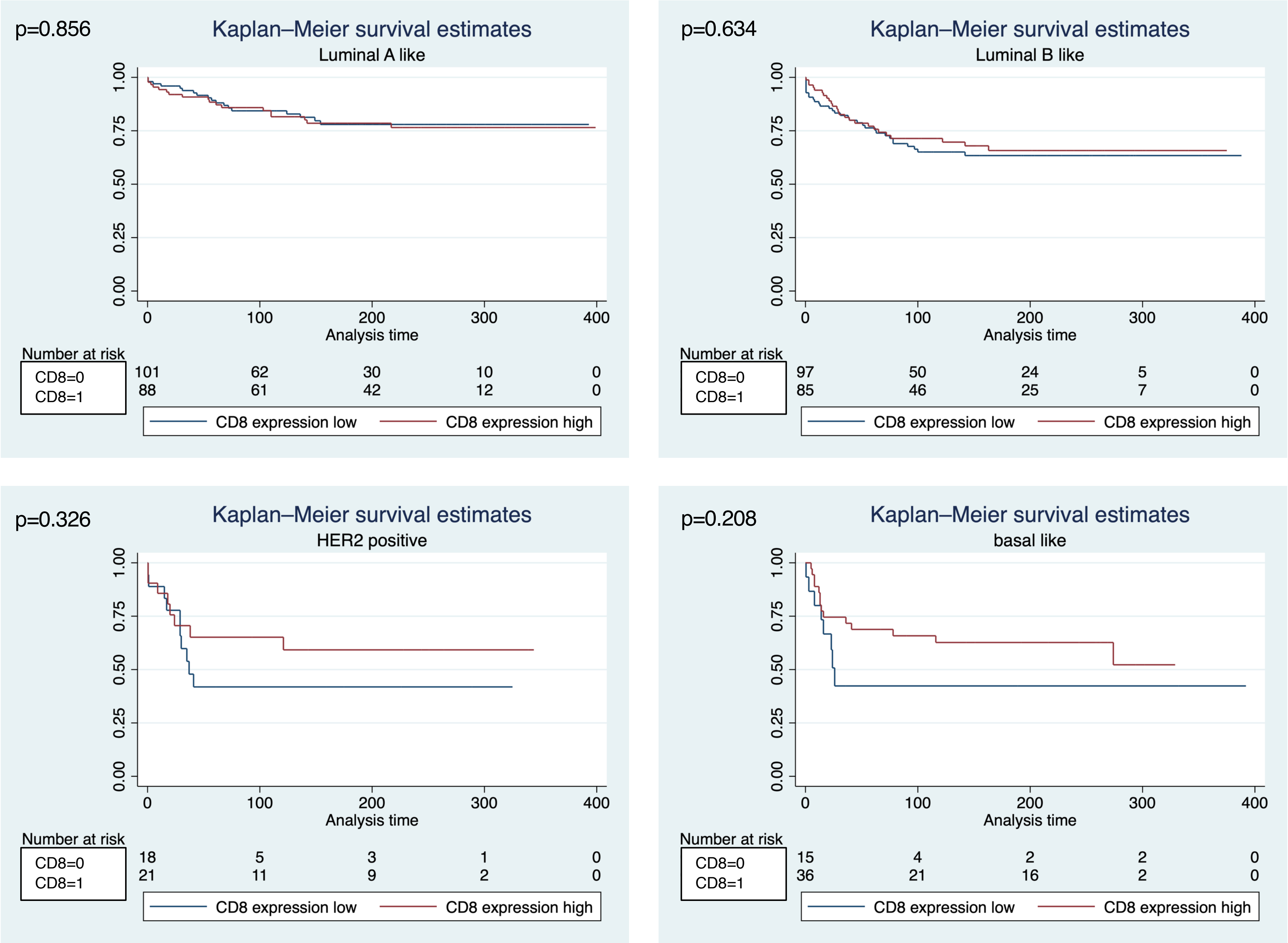
p=0.856
Kaplan–Meier survival estimates
Luminal A like
0.00 0.25 0.50 0.75 1.00
0 100 200 300 400
Analysis time
Number at risk
CD8=0 101 62 30 10 0
CD8=1 88 61 42 12 0
CD8 expression low
CD8 expression high
p=0.634
Kaplan–Meier survival estimates
Luminal B like
0.00 0.25 0.50 0.75 1.00
0 100 200 300 400
Analysis time
Number at risk
CD8=0 97 50 24 5 0
CD8=1 85 46 25 7 0
CD8 expression low
CD8 expression high
p=0.326
Kaplan–Meier survival estimates
HER2 positive
0.00 0.25 0.50 0.75 1.00
0 100 200 300 400
Analysis time
Number at risk
CD8=0 18 5 3 1 0
CD8=1 21 11 9 2 0
CD8 expression low
CD8 expression high
p=0.208
Kaplan–Meier survival estimates
basal like
0.00 0.25 0.50 0.75 1.00
0 100 200 300 400
Analysis time
Number at risk
CD8=0 15 4 2 2 0
CD8=1 36 21 16 2 0
CD8 expression low
CD8 expression high
