# Supplementary material for: Prognostic Impact of LAG-3 mRNA Expression in Early Breast Cancer
Source: Biomedicines. 2022 Oct 21;10(10):2656. doi: 10.3390/biomedicines10102656 (PMC9599264; doi:10.3390/biomedicines10102656)
Supplement: Supplementary file 1 [file biomedicines-10-02656-s001.zip › Table S1.pdf]

univariat		HR	95% CI<br>lower	 upper	p- value
**CTLA-4**	High vs. Low expression	1.078	0.767	1.515	0.666

multivariat		HR	95% CI<br>lower	 upper	p- value
**CTLA-4**	High vs. Low	0.849	0.553	1.302	0.453
**Age**	<50 vs. ≥50	1.142	0.659	1.978	0.636
**Tumor size**	T2-4 vs. T1	1.558	0.978	2.483	0.062
**Lymph node status**	N1,2,3 vs. N0	1.406	0.908	2.176	0.126
**Grade**	GIII vs. GI/II	2.188	1.385	3.458	0.001
**Ki 67**	>20% vs. <20%	1.352	0.854	2.139	0.198
