# Supplementary figures and images for: Prognostic Impact of LAG-3 mRNA Expression in Early Breast Cancer
Source: Biomedicines. 2022 Oct 21;10(10):2656. doi: 10.3390/biomedicines10102656 (PMC9599264; doi:10.3390/biomedicines10102656)

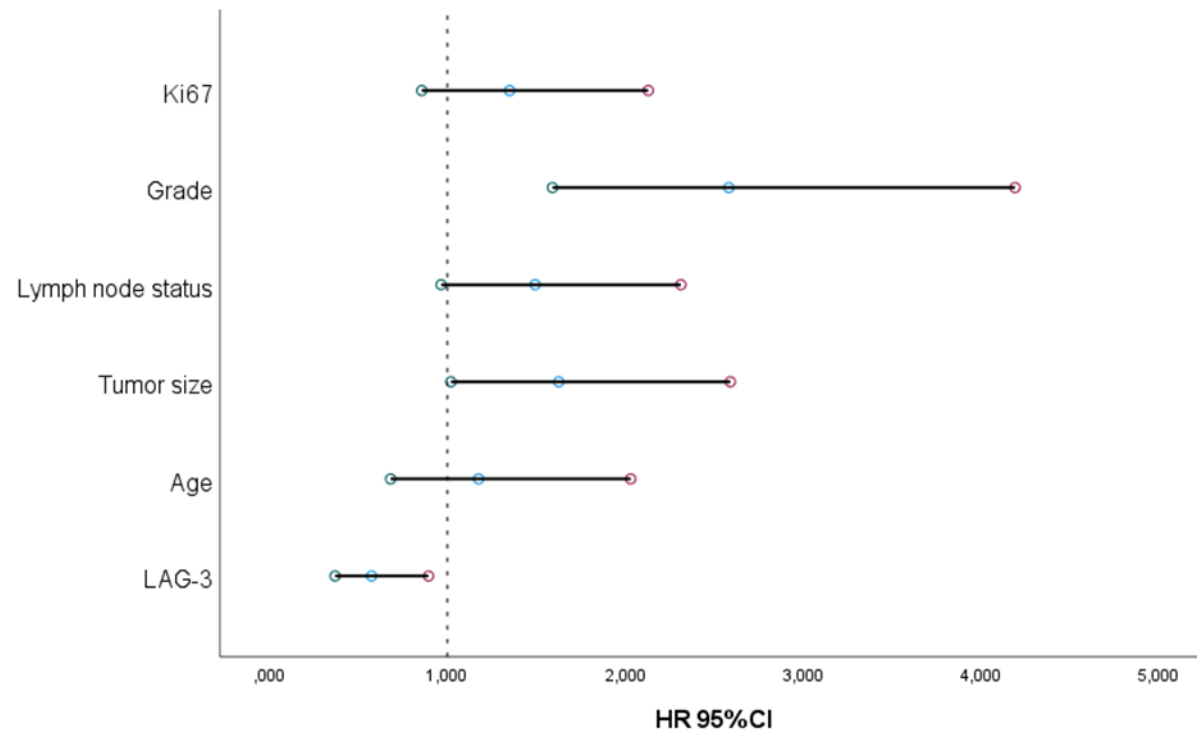

HR (95% CI); p-value

1.350 (0.856-2.130); p=0.197

2.583 (1.591-4.192); **p<0.001**

1.494 (0.965-2.313); p=0.071

1.626 (1.020-2.591); **p=0.041**

1.176 (0.681-2.031); p=0.561

0.574 (0.369-0.894); **p=0.014**

Supplement: Supplementary file 1 [file biomedicines-10-02656-s001.zip › Figure S1.pdf]

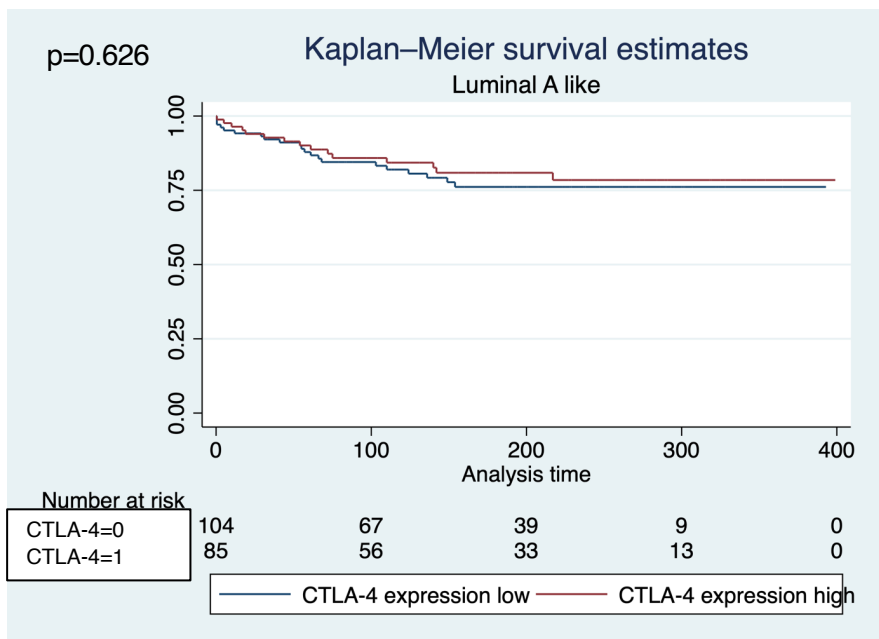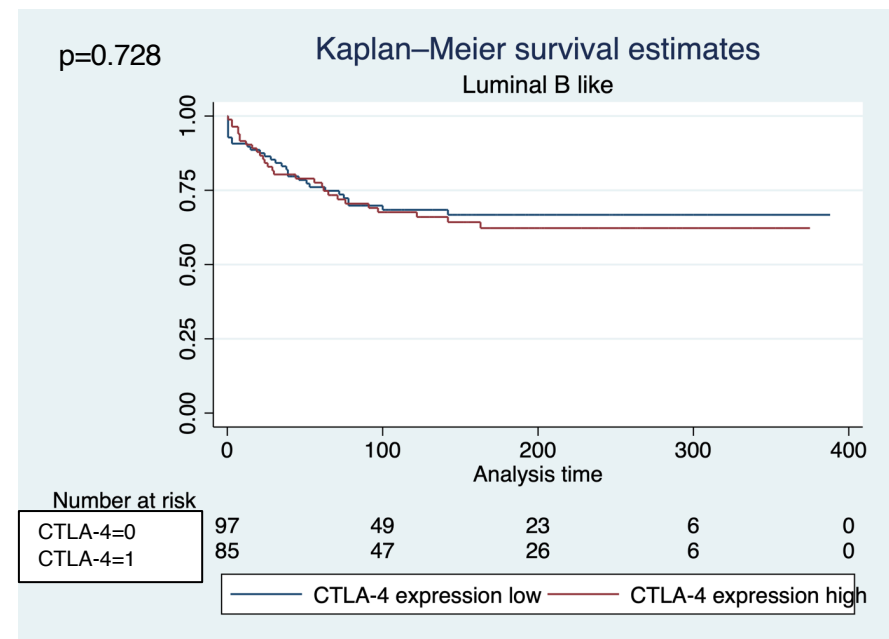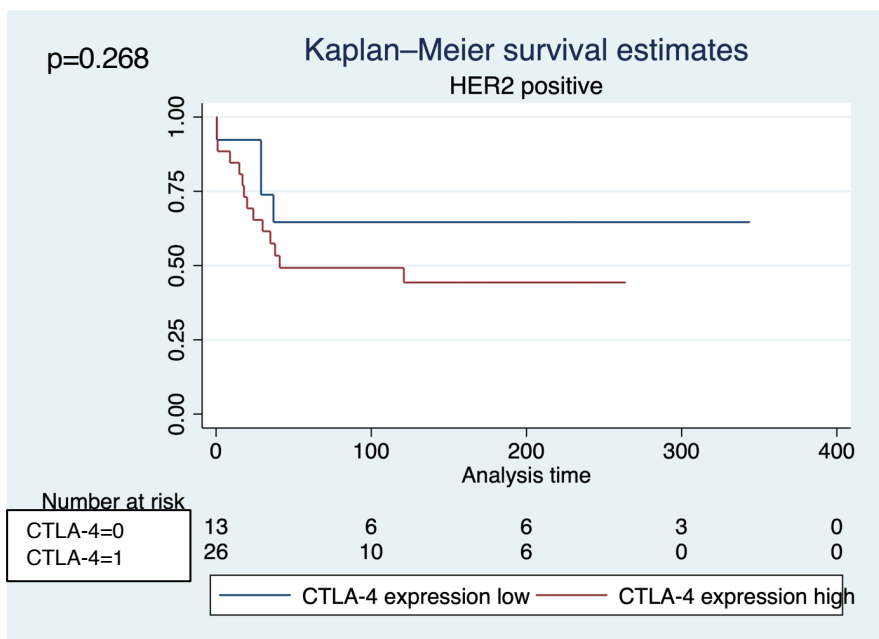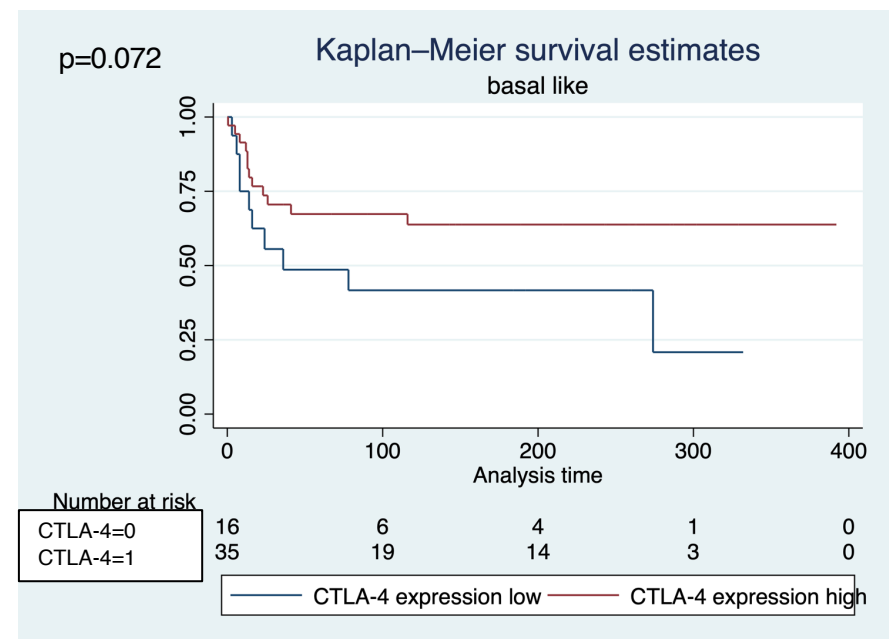

Supplement: Supplementary file 1 [file biomedicines-10-02656-s001.zip › Figure S2b.pdf]

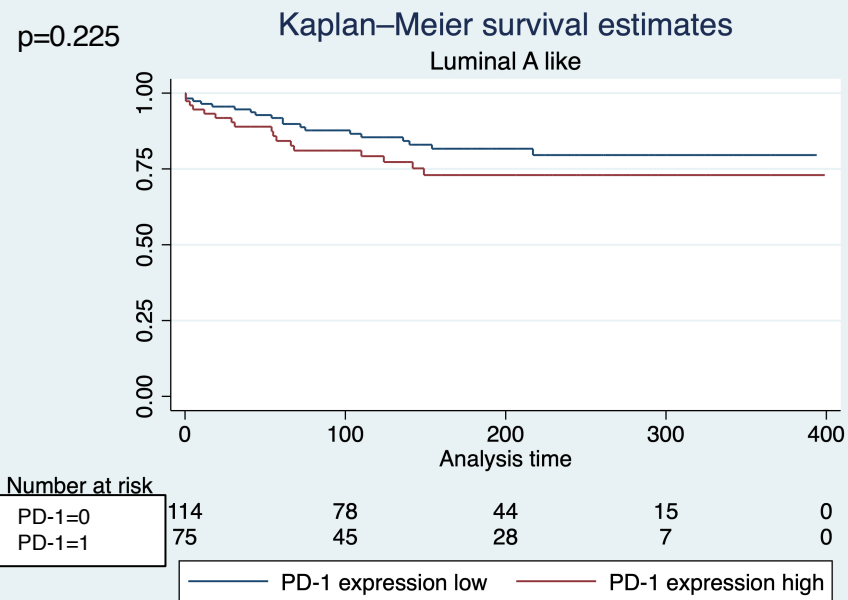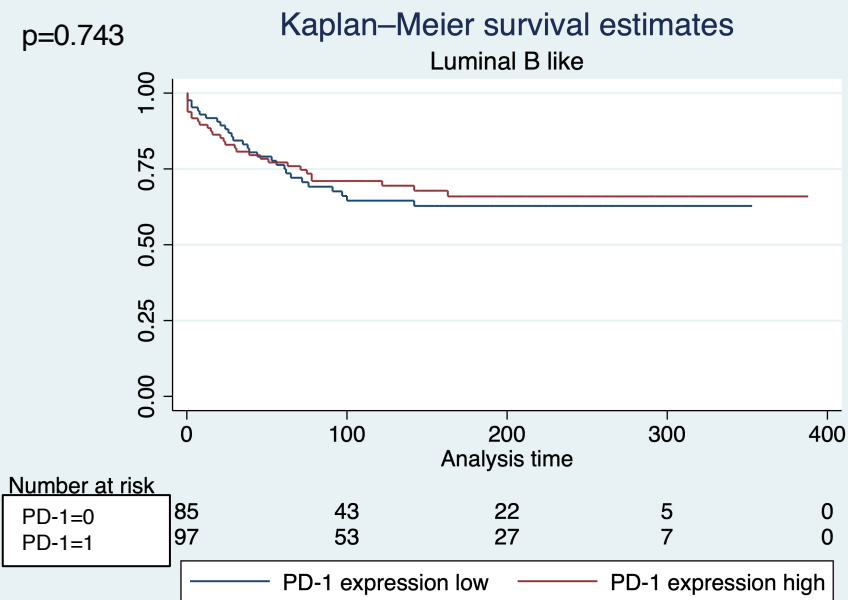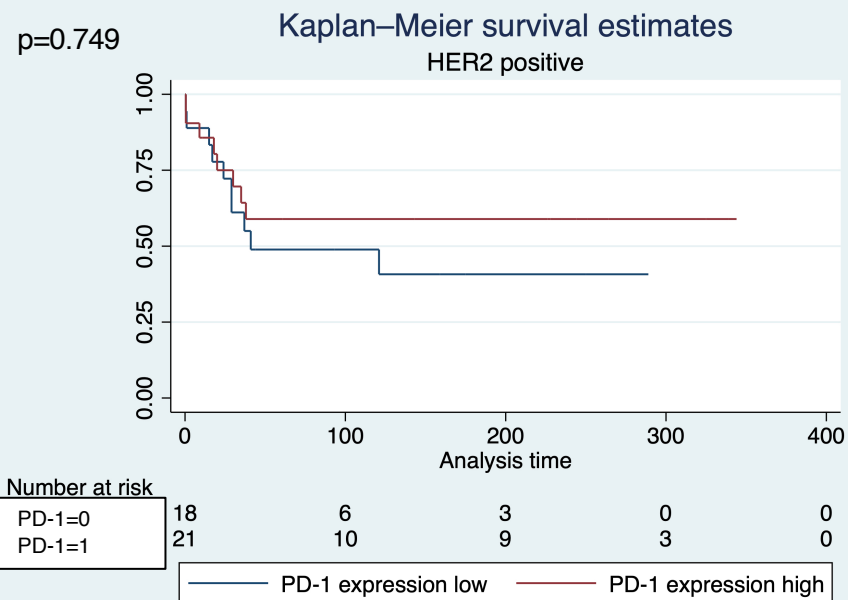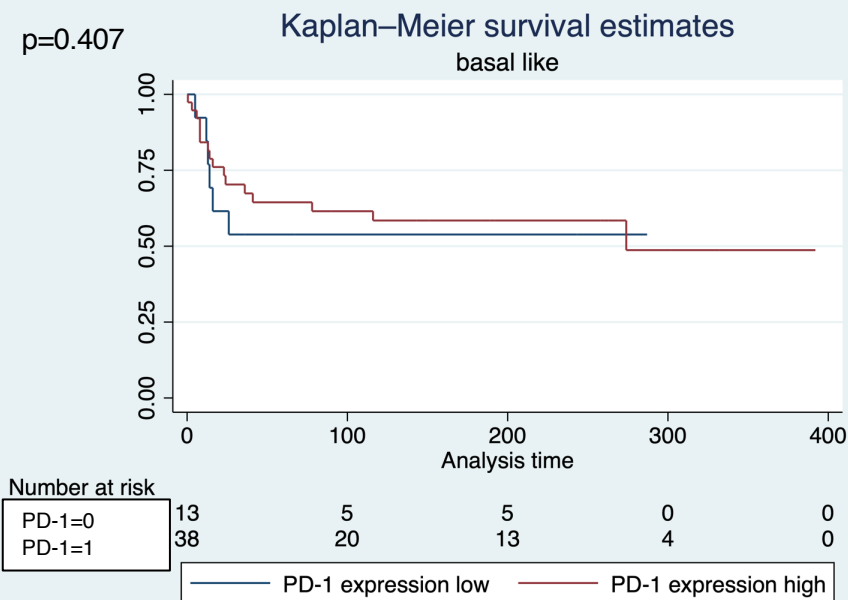

Supplement: Supplementary file 1 [file biomedicines-10-02656-s001.zip › Figure S3b.pdf]

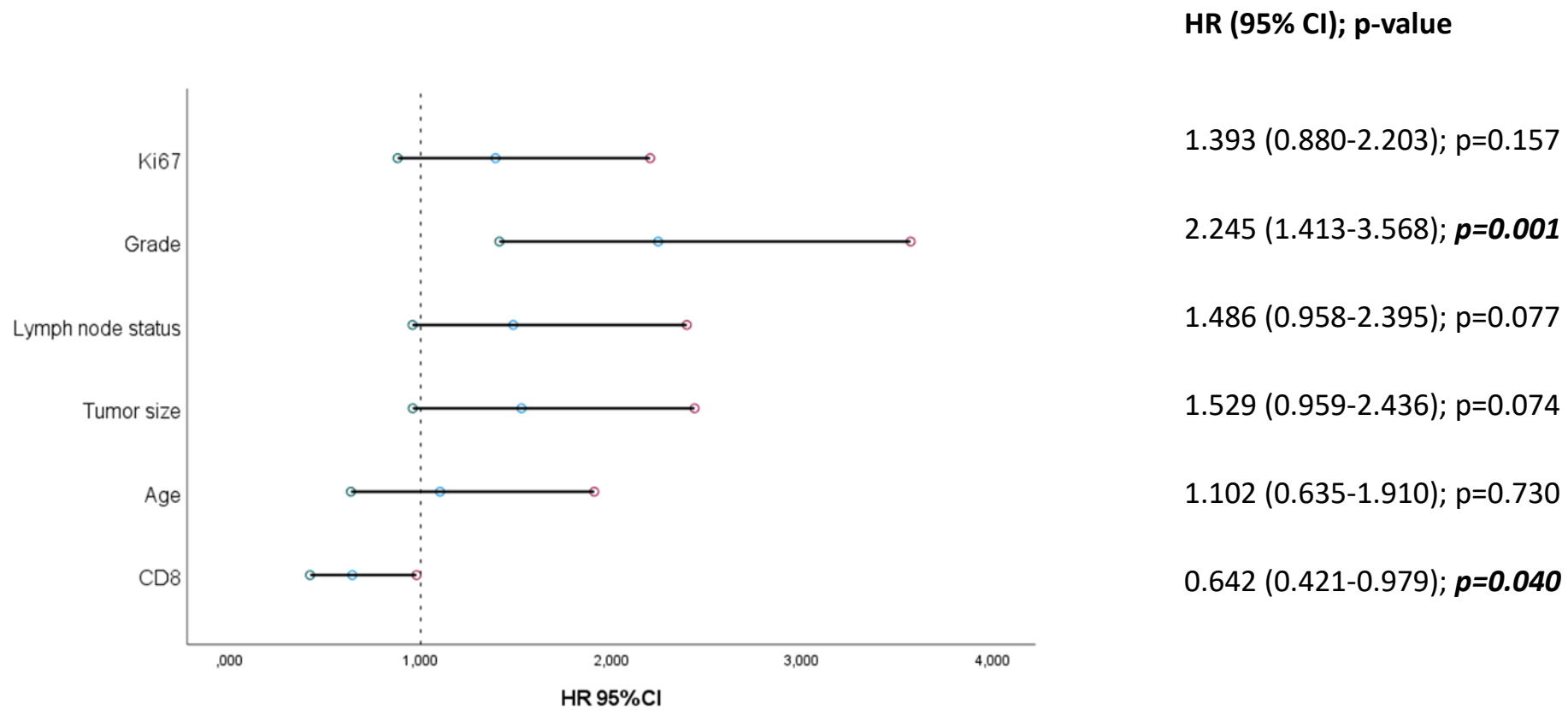

Supplement: Supplementary file 1 [file biomedicines-10-02656-s001.zip › Figure S5.pdf]

# LAG3 (206486\_at)

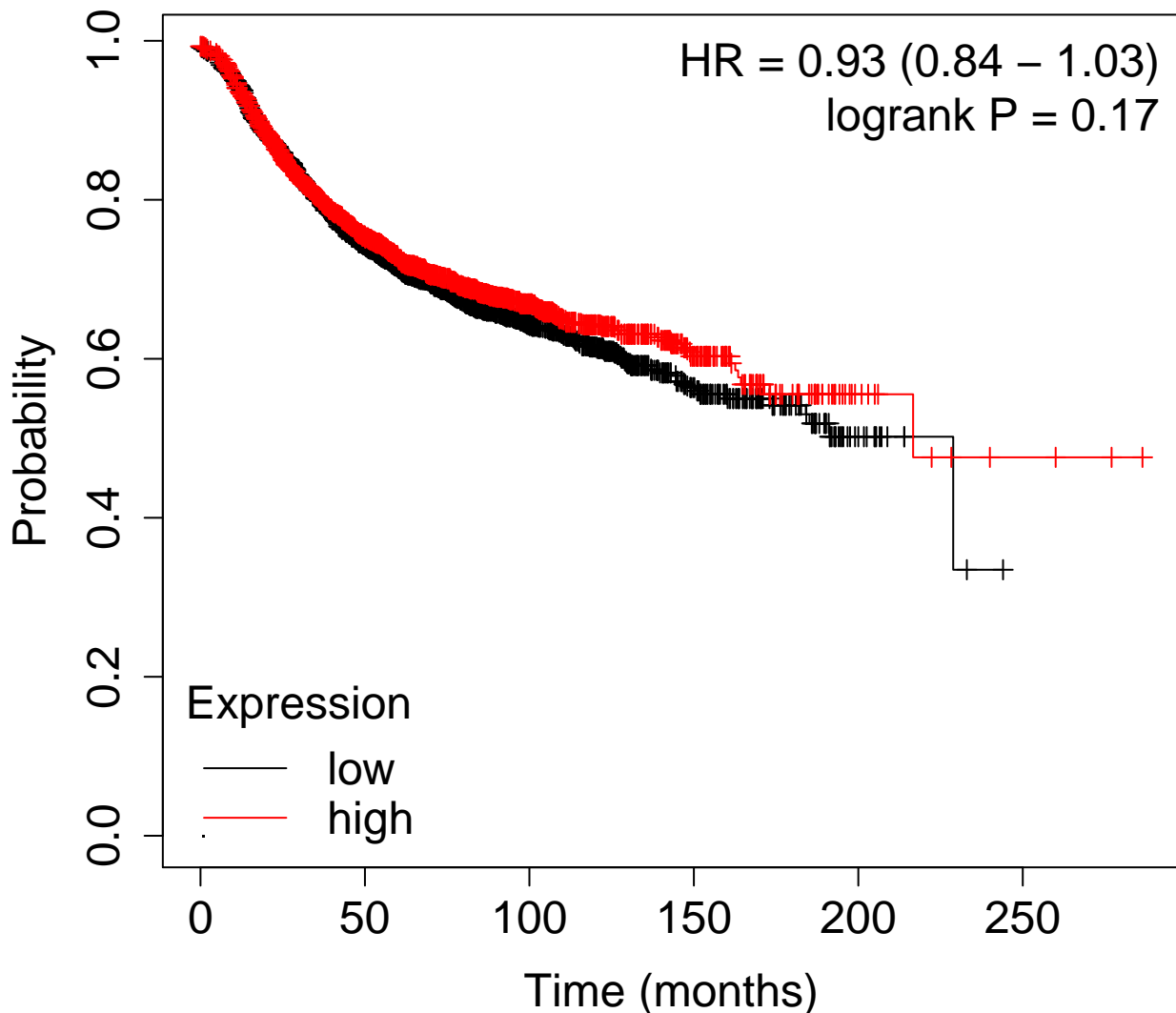

Number at risk

|      |      |      |     |     |    |   |
|------|------|------|-----|-----|----|---|
| low  | 2467 | 1431 | 569 | 138 | 15 | 0 |
| high | 2462 | 1452 | 567 | 108 | 12 | 3 |

Supplement: Supplementary file 1 [file biomedicines-10-02656-s001.zip › Figure S6a.pdf]

# LAG3 (206486\_at)

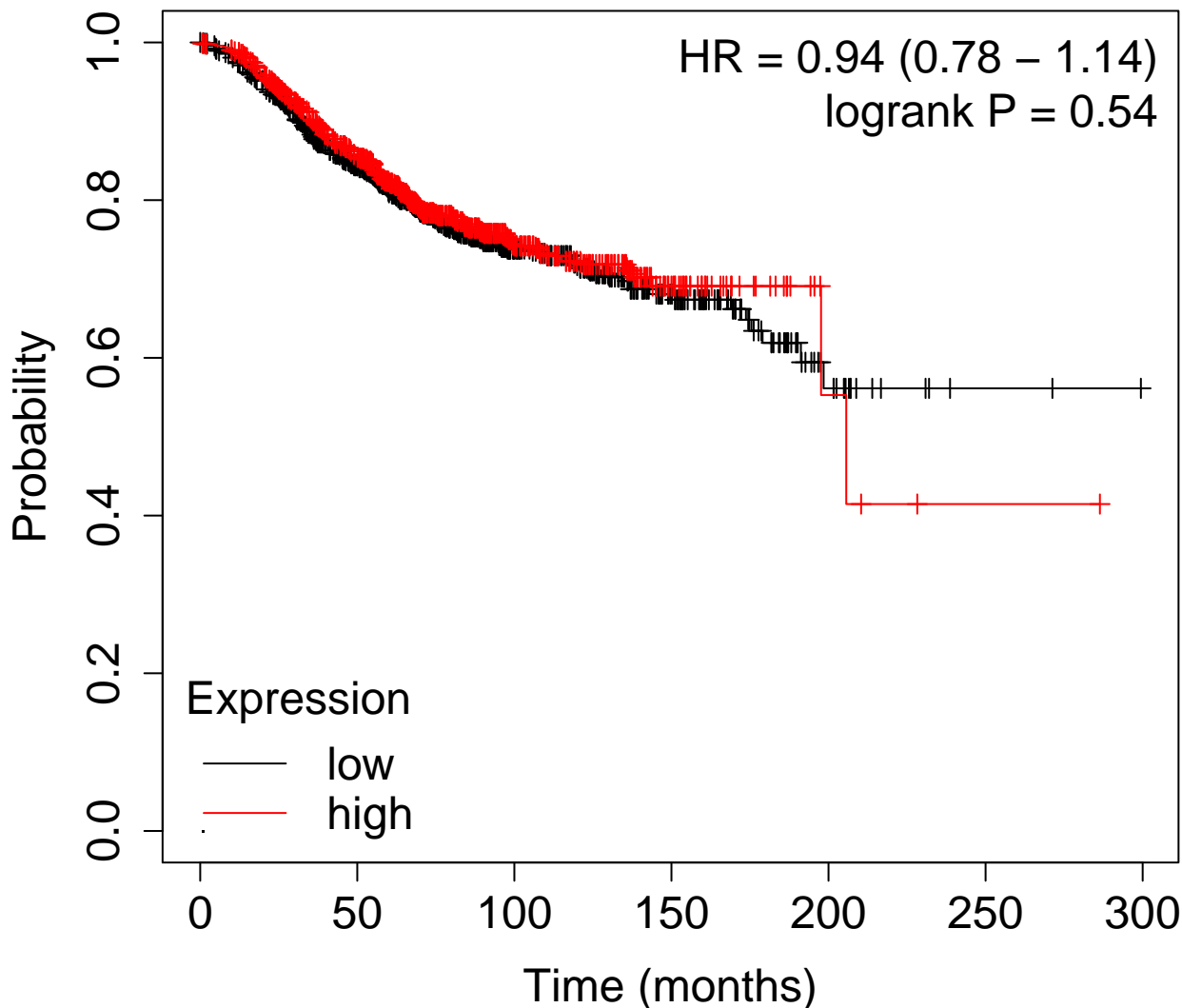

Number at risk

|      |     |     |     |    |    |   |   |
|------|-----|-----|-----|----|----|---|---|
| low  | 940 | 676 | 282 | 96 | 17 | 2 | 0 |
| high | 939 | 690 | 271 | 47 | 4  | 1 | 0 |

Supplement: Supplementary file 1 [file biomedicines-10-02656-s001.zip › Figure S6b.pdf]

# LAG3 (206486\_at)

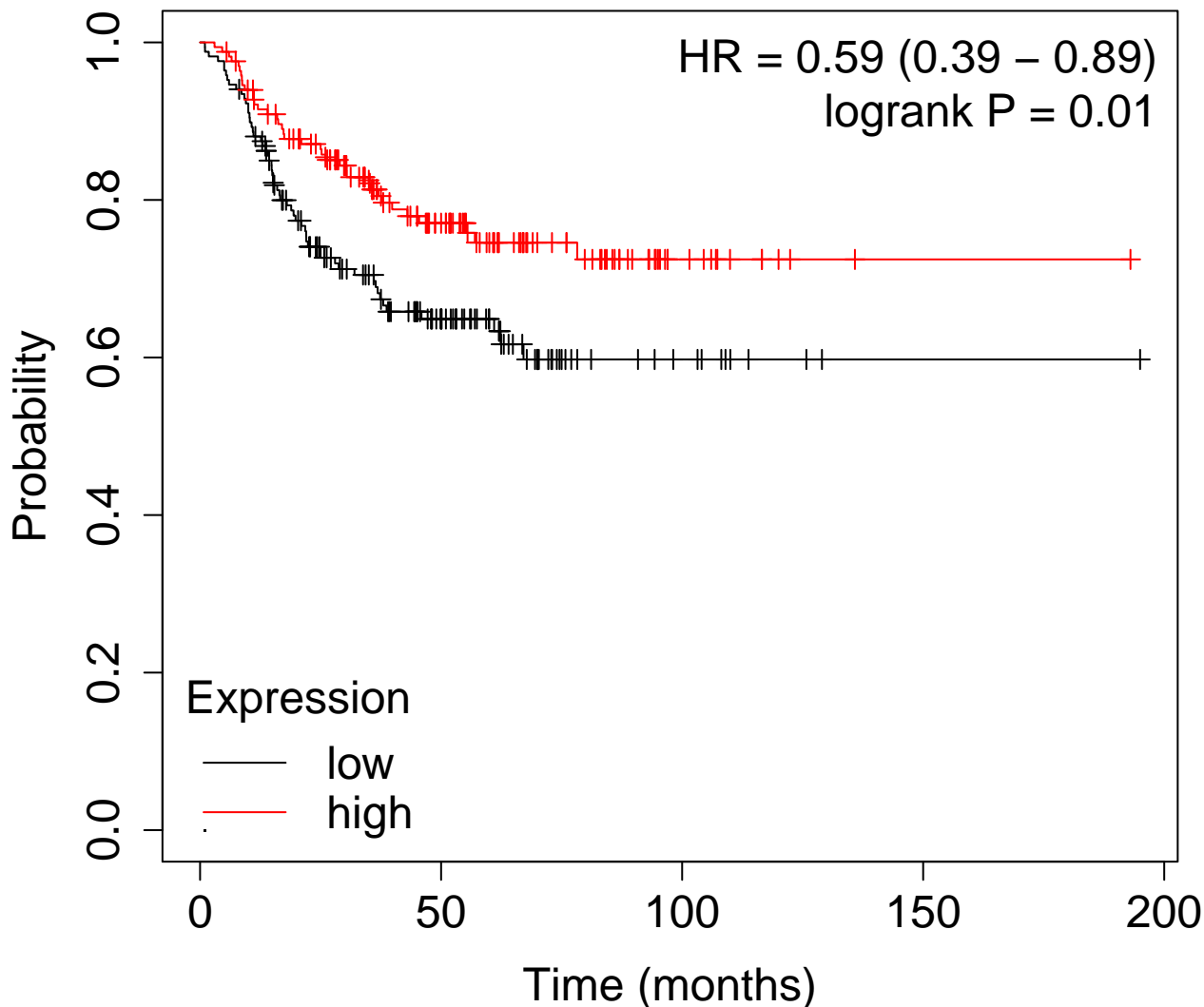

Number at risk

|      |     |    |    |   |   |
|------|-----|----|----|---|---|
| low  | 168 | 62 | 10 | 1 | 0 |
| high | 167 | 79 | 12 | 1 | 0 |

Supplement: Supplementary file 1 [file biomedicines-10-02656-s001.zip › Figure S7a.pdf]

# LAG3 (206486\_at)

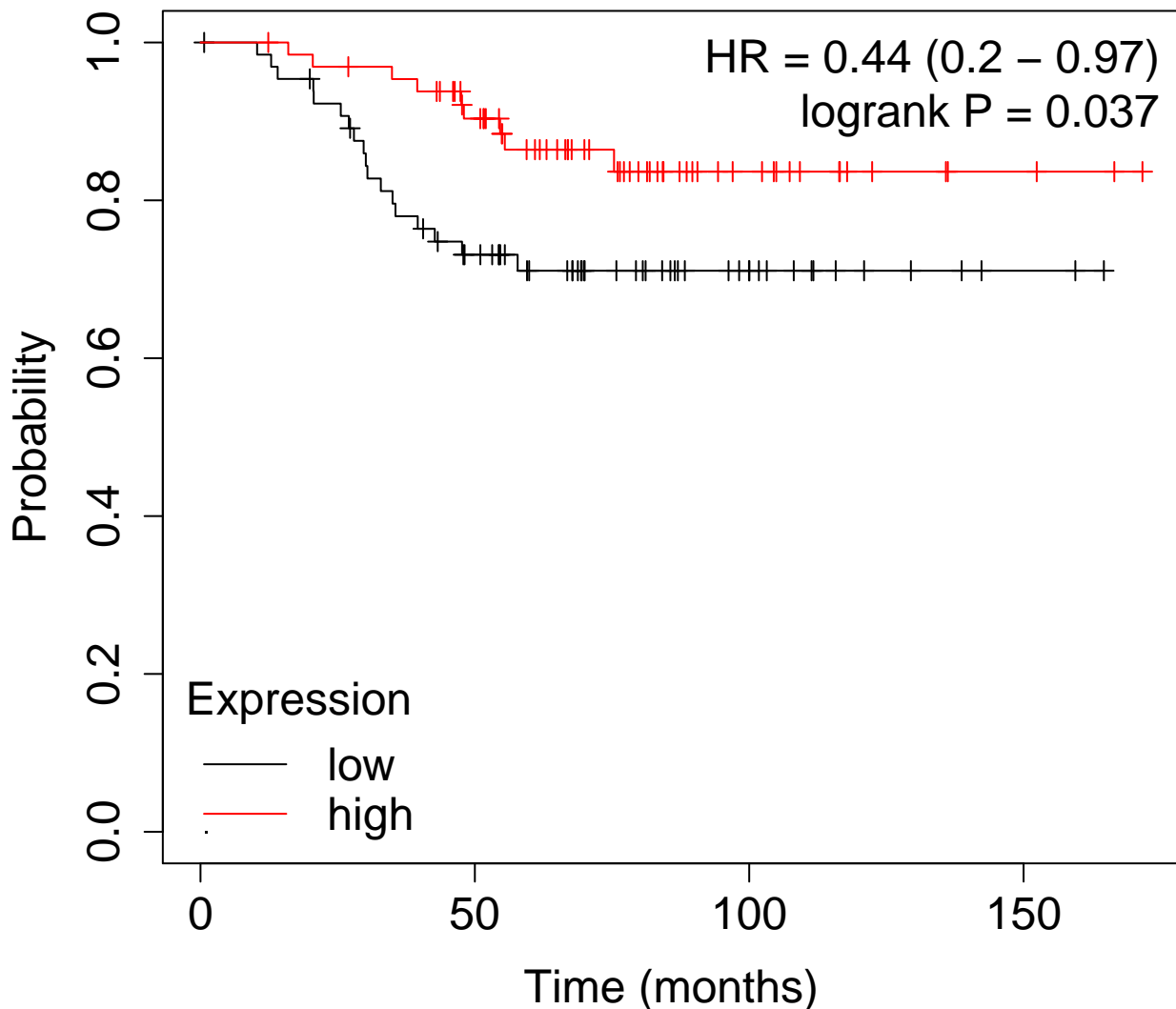

Number at risk

low  
high

66  
66

42  
52

13  
14

2  
3

Supplement: Supplementary file 1 [file biomedicines-10-02656-s001.zip › Figure S7b.pdf]
